# A simulation-based nursing education of psychological first aid for adolescents exposed to hazardous chemical disasters

**DOI:** 10.1186/s12909-022-03164-6

**Published:** 2022-02-11

**Authors:** Hye-won Kim, Yun-Jung Choi

**Affiliations:** 1 Asan Medical Center, Seoul, South Korea; 2 Red Cross College of Nursing, Chung-Ang University, Seoul, South Korea

**Keywords:** Adolescent, Disaster, Hazardous chemicals, Nursing education, Psychological first aid, Simulation

## Abstract

**Background:**

Simulation-based education facilitates a learner-centered experience, which has been found to be effective in improving clinical performance, problem-solving ability, and self-confidence in nursing practice. The objective of this study was to develop and test a psychological first aid simulation-based education program for nurses caring for adolescents exposed to hazardous chemical disasters.

**Methods:**

This study employed a nonequivalent pre and post-control group research design. The simulation-based education program was developed, and the participants were 30 nurses working in a medical center who were randomly assigned to the experimental, comparison, and control groups. The collected data were statistically analyzed using IBM SPSS Statistics for Windows, Ver. 22.0.

**Results:**

The nurses who participated in the simulation-based education program showed statistically significantly improved psychological first aid performance knowledge, competence, and self-efficacy compared to those in the other groups.

**Conclusions:**

Nursing simulation programs could help to improve nurses’ performance in mental health care and psychological support for adolescents suffering from hazardous chemical disasters.

## Introduction

Exposure to hazardous chemicals can cause respiratory symptoms, such as pneumonia, pulmonary edema, and pulmonary thrombosis, as well as other ailments, including headaches and neurological symptoms accompanied by permanent brain damage and gastrointestinal disorders [1]. Unfortunately, even after these physical symptoms have been relieved, many victims still show psychological trauma. In fact, an evaluation of the psychological status of 1300 victims of the hydrogen fluoride leak found that 374 were suffering from post-traumatic stress disorder (PTSD) and anxiety disorder [2]. If the initial response to these psychological problems is delayed, the sufferers may experience long-term effects, such as suicide, alcoholism, domestic violence, drug addiction, and depression, which can affect the social fabric of their community [3, 4].

In particular, adolescents lack coping skills and require more psychological support for recovery compared to adults because they are in the process of cognitive, emotional, and social development. Adolescents experience greater difficulties than adults in response to psychological trauma, such as painful physical symptoms and flashbacks, which are sometimes expressed as aggressive behaviors [5]. Traumatic experiences in adolescence can lead to various mental health problems in adulthood, including impulsive and aggressive behaviors, self-harm, eating disorders, dissociative disorders, and personality disorders [6]. Therefore, nurses in clinical settings must care for the psychological state of adolescent victims from the initial impact of the disaster through to recovery. To this end, a mental health education program for nurses providing psychological support for adolescent disaster victims needs to be developed [5].

The Psychological First Aid (PFA) program provided by the World Health Organization (WHO) is recognized as an effective way to guide the psychological support activities of disaster mental health professionals, including nurses [7]. The goal of PFA is to intervene early in a disaster situation to alleviate the impact of psychological trauma and to help the victims adapt to daily life by promoting functional recovery [8].

Most of the PFA educational methods are theoretical and role-play oriented, but simulation-based education using standardized patients (SPs), which has been in the spotlight recently, is similar to real life and enables indirect experiences in a safe clinical environment reproduced through scenarios. Therefore, it provides an effective method of nurse education [9]. In addition, simulation education using SPs facilitates a learner-centered nursing experience, which has been found to be effective in improving clinical performance, problem-solving ability, and self-confidence [10]. Therefore, providing nurses with various simulated training opportunities related to disaster psychological support strengthens their disaster response capabilities [11].

A guiding question for this study included “How to increase nurses’ PFA performance for adolescents exposed to hazardous chemical disasters by an effective education?” Therefore, the objective of this study was to develop and test a PFA simulation-based education program for nurses caring for adolescents exposed to hazardous chemical disasters. The specific goals were as follows: (1) develop a simulation-based adolescent PFA education program using SPs and (2) verify the effect of the program on nurses’ PFA performance knowledge, competence, and self-efficacy.

## Methods

### Study design

This study was experimental research that applied a nonequivalent pretest-posttest control group design (Table 1).

**Table 1:** Study Design

Group	Pretest	Treatment	Posttest
Experimental (*n* = 10)	*e1*	Simulation-based Education	*e2*
Comparison (*n* = 10)	*cp1*	Conventional Lecture	*cp2*
Control (*n* = 10)	*ct1*	Handout	*ct2*

### Participants

The participants of this study were clinical nurses working at a general hospital in Seoul who were interested in disaster mental health nursing. They indicated their intention to participate in the study and voluntarily signed a consent form. The minimum sample size was calculated with a significance level of .05, a power of .80, and an effect size of .40 using the G-power 3.1.2 program. A total of 30 participants were selected using convenience sampling.

### Simulation

The simulation was conducted at a nursing simulation center in a university in Seoul; it included pre-briefing, scenario running, and debriefing stages (Table 2).

**Table 2:** Simulation-based Education Program

Category	Content	Educator: Learner	Time (Min)	
Lecture	Mental health care of adolescents in hazardous chemical disasters	1:10	100	
Simulation	Pre-briefing	1: 5	8	50
	Simulation running with a standardized patient	1: 1	12	
	Debriefing	1: 2	30	
Self-directed learning	Learning materials	–	90	
Total			240	

The simulation scenario started with a nurse facing a female patient complaining of anxiety after evacuation due to a disaster in which hazardous chemicals were leaked. The learner provided one-on-one psychological support and nursing care to the SP for 12 min. While waiting for the next step of the simulation, participants were encouraged to engage in self-directed learning using a workbook on disaster mental health nursing and psychological support in a separate room.

The 30-min debriefing sessions involved one instructor and two learners and were conducted based on the SENSE (Sharing-Explore-Notice-Support-Expansion) model [12], which includes learner stress management and emotional support related to simulation learning. The entire simulation operation was conducted by the researcher and five research assistants.

### Instruments

#### Psychological first aid performance knowledge

PFA performance knowledge was measured using Park and Choi [4] ‘s tool, which modified Lee et al. [7]‘s PFA knowledge measurement tool to apply it to disaster mental health research. This tool consists of a total of 10 yes (=1) or no (=0) to the questions, with a higher score indicating greater PFA performance knowledge. The Kuder-Richardson Formula 20 (KR-20) was used to calculate the reliability of the tool, and it was found to be KR-20 = .76.

#### Psychological first aid performance competence

PFA performance competence was measured using Park and Choi [4]‘s tool, a modified version of Noh [13]‘s disaster nursing core performance measurement tool based on the International Nursing Association’s Disaster Nursing Performance Competence Scale. This tool is a 5-point Likert scale consisting of a total of 12 items with options ranging from 1 point “not at all” to 5 points “strongly agree,” with a higher score indicating greater PFA performance competence. The reliability of the tool was found to be Cronbach’s α = .94 in Noh [13]‘s study and Cronbach’s α = .90 in this study.

#### Psychological first aid performance self-efficacy

To measure PFA performance self-efficacy, the General Self-Efficacy Scale [14] modified by Jung [15] and supplemented with the Korean version by Lee [16] was used. This tool is a 5-point Likert scale consisting of a total of 16 items with options ranging from 1 point “not at all” to 5 points “strongly agree,” with higher scores indicating higher self-efficacy. The reliability of the tool was found to be Cronbach’s α = .95 in Lee [16]‘s study and Cronbach’s α = .91 in this study.

### Data collection

The data collection period was from January 15 to February 6, 2019. The study participants were asked to respond to a self-reported questionnaire before and after the experimental treatment. It took about 15 min to respond to the entire questionnaire, and the completed questionnaires were sealed and stored in a confidential location after coding for data analysis.

### Data analysis

The data of this study were analyzed using IBM SPSS Statistics for Windows, Version 22.0. The general characteristics of the participants were analyzed using descriptive statistics. Homogeneity between groups was analyzed using the Chi-square test and Fisher’s exact test. One-way ANOVA was used to verify differences in PFA knowledge, PFA performance competence, and self-efficacy of the participants.

### Ethical considerations

This study was approved by the Chung-Ang University Institutional Review Board (No.1041078–201,808-HRSB-167-01C). The study participants voluntarily signed the informed consent form after receiving a detailed explanation of the research purpose and method. It was explained to the participants that the questionnaire answers would be kept confidential and anonymous and that they would not be used for any purpose other than the intended research. The participants were informed that they could withdraw from the study at any time and that there would be no disadvantage in doing so.

## Results

The number of participants was 30 in total, 10 in each of the experimental, comparison, and control groups. Among them, women accounted for the majority (93.3%), and the average age was 29 years. Among the study participants, 6.7% of nurses had experience in caring for disaster victims, meaning that most of them had no experience in disaster nursing. The study participants were found to be homogeneous among the three groups, as there was no statistically significant difference in any of the general characteristic items (Table 3).

**Table 3:** General Characteristics and Homogeneity of Participants

Characteristics	Exp. (***n*** = 10)	Comp. (***n*** = 10)	Cont. (***n*** = 10)	X^**2**^/F(***p***)
	Frequency (%)			
Age (M ± SD)	29.00 ± 2.625	28.20 ± 4.104	31.10 ± 6.082	1.108 (.345)
Gender				
Female	10 (100)	9 (90)	9 (90)	1.312 (1.000)
Male	0 (0)	1 (10)	1 (10)	
Marital status				
Unmarried	7 (70)	9 (90)	6 (60)	2.372 (.450)
Married	3 (30)	1 (10)	4 (40)	
Education				
Bachelor	10 (100)	10 (100)	10 (100)	.
Religion				
Christian	2 (20)	3 (30)	2 (20)	3.097 (.938)
Catholic	2 (20)	1 (10)	3 (30)	
Buddhist	1 (10)	0 (0)	1 (10)	
None	5 (50)	6 (60)	4 (40)	
Working position				
General nurse	10 (100)	9 (90)	8 (80)	2.033 (.754)
Charge nurse	0 (0)	1 (10)	2 (20)	
Working period				
< 3 years	3 (30)	7 (70)	2 (20)	6.109 (.185)
3–5 years	1 (10)	1 (10)	2 (20)	
> 5 years	6 (60)	2 (20)	6 (60)	
Working unit				
Internal medicine ward	5 (50)	2 (20	0 (0)	12.225 (.070)
Surgical ward	1 (10)	6 (60)	5 (50)	
Pediatric ward	0 (0)	0 (0)	1 (10)	
Special unit	3 (30)	1 (10)	3 (30)	
Full-time unit	1 (10)	1 (10)	1 (10)	
Employment type				
Full-time	1 (10)	1 (10)	1 (10)	
Three shifts	8 (80)	9 (90)	9 (90)	2.329 (1.000)
Other	1 (10)	0 (0)	0 (0)	
Disaster work experience				
Yes	0 (0)	2 (20)	0 (0)	2.909 (.310)
No	10 (100)	8 (80)	10 (100)	

The effect of the disaster mental health nursing simulation on the PFA performance knowledge of nurses caring for adolescent patients exposed to hazardous chemicals was analyzed (Table 4). The average score of the experimental group increased from 3.80 points to 9.50 points, while that of the comparison group increased from 4.30 points to 8.40 points and that of the control group increased from 4.30 points to 4.90 points, indicating significant differences between the three groups (F = 29.917, *p* < .001).

**Table 4:** Effects of the Simulation-based Education (*N* = 30)

Variable	Pretest (M ± SD)	Posttest (M ± SD)	F or z	***p***
PFA Performance Knowledge				
Exp. (*n* = 10)	3.80 ± 1.229	9.50 ± .850	29.917	<.001^***^
Comp. (*n* = 10)	4.30 ± 1.160	8.40 ± 1.075		
Cont. (*n* = 10)	4.30 ± 1.160	4.90 ± 1.197		
PFA Performance Competence				
Exp. (*n* = 10)	25.50 ± 7.878	51.80 ± 4.104	38.448	<.001***
Comp. (*n* = 10)	24.70 ± 6.464	36.60 ± 9.879		
Cont. (*n* = 10)	26.40 ± 4.551	31.80 ± 6.215		
PFA Performance Self-efficacy				
Exp. (*n* = 10)	54.60 ± 6.450	69.90 ± 3.635	13.517	.001^**^
Comp. (*n* = 10)	53.20 ± 9.355	57.60 ± 10.690		
Cont. (*n* = 10)	51.60 ± 6.899	52.30 ± 8.433		

^*^p < .05, ^**^p < .01, ^***^p < .001

The effect of the disaster mental health nursing simulation on PFA performance competence was then analyzed. The average score of the experimental group increased from 25.50 points in the pretest to 51.80 points in the post-test, whereas that of the comparison group increased from 24.70 points to 36.60 points and that of the control group increased from 26.40 points to 31.80 points, showing significant differences between the three groups (F = 38.448, *p* < .001).

Finally, the effect of the disaster mental health nursing simulation on PFA performance self-efficacy was analyzed. The average score of the experimental group increased from 54.60 points to 69.90 points, that of the comparison group increased from 53.20 points to 57.60 points, and that of the control group increased from 51.60 points to 52.30 points, indicating significant differences among the three groups (z = 13.517, *p* = .001).

## Discussion

This study developed a disaster simulation-based education for nurses providing psychological support to adolescents and evaluated its effects on PFA performance knowledge, competency, and self-efficacy.

In this study, the PFA performance knowledge improved in the experimental group participating in the simulation-based education compared to the other groups, and there was a statistically significant difference. This result is consistent with the previous studies that improved PFA performance knowledge by applying simulation-based education programs for relief workers [10] and emergency room nurses [17]. Therefore, it is necessary to increase PFA performance knowledge by providing simulation-based education to nurses to provide adequate psychological support to clients affected by disasters [11].

The PFA simulation-based education of this study showed statistically higher PFA performance competence than the other groups, indicating a significant difference. This result is in line with previous research that simulation-based education improves PFA performance competence for nurses in caring for victims of a fire disaster [4]. This simulation-based education would improve PFA performance competence by combining knowledge and skills for psychological support in a realistic environment [18–20].

In the nursing care of adolescents, applying simulation education using SPs is more effective in improving PFA performance self-efficacy of nurses [21]. Thus, in this study, the nurses who participated in the PFA simulation-based education using an SP showed statistically significant differences from the other groups who only received theoretical education and individualized handout-based learning. This result is consistent with a study that improved PFA performance self-efficacy by using an SP for mental health practitioners [4].

This study developed and applied simulation-based education for nurses to support the psychological rehabilitation of adolescents exposed to a hazardous chemical disaster. The simulation-based education in this study would help nurses increase PFA performance in disasters and crises and contribute to promoting mental health in the community by ensuring early intervention for adolescents.

This study contributes to providing evidence-based education, including PFA training materials for nurses in psychological support of adolescents exposed to chemical disasters. The education developed in this study will contribute nurses to effectively providing psychological support for adolescents after disasters in nursing practice. This study would contribute to research that facilitates further research to develop various simulation-based educations for nurses and other professionals in disaster psychological support and explore the effect.

## Conclusions

This study confirmed that the disaster simulation-based education for nurses caring for adolescents exposed to hazardous chemicals effectively improves nurses’ PFA performance knowledge, competence, and self-efficacy. This study is meaningful because it developed and verified simulation-based education for nurses who provide nursing care, including psychological support for adolescents exposed to disaster. Limitations of this study include that the research participants were recruited by convenience sampling, and due to the small number of participants, it is necessary to be cautious in interpreting and generalizing the results.

## Data Availability

The data and materials are available from the corresponding author on reasonable request.
